# A novel analysis of heat transfer in the nanofluid composed by nanodimaond and silver nanomaterials: numerical investigation

**DOI:** 10.1038/s41598-021-04658-x

**Published:** 2022-01-25

**Authors:** Adnan, Umar Khan, Naveed Ahmed, Syed Tauseef Mohyud-Din, M. D. Alsulami, Ilyas Khan

**Affiliations:** 1 Department of Mathematics, Mohi-Ud-Din Islamic University, Nerian Sharif AJ&K, AJK, 12080 Pakistan; 2 Department of Mathematics and Statistics, Hazara University, Mansehra, 21120 Pakistan; 3 Department of Mathematics Faculty of Sciences, HITEC University, 47070, Taxila Cant, Pakistan; 4 Department of Mathematics, College of Sciences and Arts at Alkamil, University of Jeddah, Jeddah, Saudi Arabia; 5 Department of Mathematics, College of Science Al-Zulfi, Majmaah University, Al-Majmaah, 11952 Saudi Arabia; 6 University of Multan, Multan, 66000 Pakistan

**Keywords:** Engineering, Mathematics and computing, Nanoscience and technology

## Abstract

The investigation of thermal performance in the nanofluids for unsteady boundary layer flow by considering the impacts of suction/injection is a significant research area in the field of fluid dynamics. This flow situation is broadly used in aerodynamics and space sciences as well. The model is formulated for ND-H_2_O and Ag-H_2_O nanofluids and then tackled numerically and captured the dynamics of the nanofluids under the multiple flow parameters. From the results, it is investigated that both ND-H_2_O and Ag-H_2_O have high thermal performance characteristics. However, higher heat transfer performance is observed for Ag based nanofluid. Further, a graphical and tabular comparison under certain assumptions is provided to authenticate the analysis.

## Introduction

Thermal performance of the fluids has a paramount significance in industrial and engineering zone. To accomplish many industrial and engineering processes, huge amount of heat transfer is needed. The conventional liquids are fails to produce desired amount of heat to accomplish the process. Therefore, it was thought that how to improve the heat transfer of regular liquids. Finally, researchers and scientists of the time came up with an idea that heat transfer of the conventional liquids could be enhanced by adding metallic and non-metallic nano additives in the host liquid. This newly developed generation of the fluid is termed as Nanofluid. These fluids have very rich heat transfer characteristics and unlock the door for industrialist and engineers towards modern world. The applications of nanofluids comprised in aerodynamics, medical sciences, computer chips, paint industry, manufacturing of aircraft parts and many more. Therefore, the study of heat transport is unavoidable due to aforementioned applications.

The dynamics of boundary layer unsteady flow of nanofluids is a very interesting research area in Fluid Dynamics. Therefore, fluid dynamists tried to examine the dynamics of the fluids over an unsteady stretching surface under various flow conditions. The dynamics of the nanofluids composed by various metals namely TiO_2_, Cu, Silver and Al_2_O_3_ over an unsteady stretchable surface are examined in Elgazery^1^. They found the numerical solution of the colloidal model and captured the flow characteristics against various flow parameters involved in the study. The decrement in the fluid motion was reported by strengthening the magnetic field effects. Further, they concluded that the nanofluid comprising TiO_2_ nanomaterial is a better heat conductor. Although, the study was interesting but lake the heat transport mechanism for novel nanomaterials like Nanodiamond and silver. Their study also included the influences of suction and injection of the fluid and fascinating variations in the dynamics of nanofluids examined.

The heat transport investigation in Darcy Forchheimer nanofluid by taking unsteadiness effects in the governing model was described by Nasir et al.^2^. The flow model was handled analytically and captured the graphical results against the flow quantities. Further, they reported that the local heat exchange rate rises against higher volumetric fraction of the nanomaterial. Recently, Kebede et al.^3^ conducted a study to examine the heat and mass transport mechanism in convective boundary layer flow of Williamson fluid. They accounted the effects of permeability in the governing flow equations and treated the model analytically. The augmentation in the heat and mass transfer due to Williamson parameter was concluded in their analysis.

The transfer of heat due to thermal radiations is another heat transfer phenomenon and researchers conducted numerous studies to analyze the heat transport mechanism by imposing thermal radiation over the domain of interest. Keeping in view, Sreedevi et al.^4^ reported the dynamics of hybrid nanofluids over a stretchable surface. The study was conducted in cartesian coordinates by taking the influences of thermal radiations, slip and suction effects, and chemical reaction parameter. Moreover, augmentations in the temperature of the hybrid nanofluid were core findings of the study. Another significant analysis of the nanofluid over an unsteady stretchable surface was descried by Das et al.^5^. In ordered to increase the novelty of the study, they also plugged thermophoresis and brownian motion effects in the study. They found that the radiative nanofluids temperature enhances for higher brownian motion and declines against thermophoresis parameters over the region of interest.

The convective thermal performance in an unsteady nanofluid past a stretchable sheet was reported in Prasannakumara et al.^6^. They reported the analysis for dusty nanofluid and observed that more radiative surface is better for thermal enhancement in dusty nanofluid. Further, they authenticate the results by providing a comparative analysis under certain assumptions with the results reported in the literature. In 2014, Bhattacharyya and Layek^7^ described the nanofluids heat transfer in the presence of imposed Lorentz forces. They considered the flow situation over exponentially stretchable surface positioned in cartesian coordinates system. The problem was tackled by employing 4th order RK technique coupling with shooting algorithm. The increment in the temperature due to higher brownian motion effects and decrement against high volume fraction was major outcomes of their study.

The dynamics of the fluid flowing over a surface which has ability to stretched or shrinked are reported by Mansur and Ishak^8^. They contemplated the flow of an unsteady nature and formulated the model by imposing the convective heat condition over the surface. The solutions of the formulated model over an infinite semi-region are then attained via shooting algorithm and captured the results against the preemenant parameters in the form of graphs. They reported that the local thermal rate performance at the surface depreciates by increasing the shrinking/stretching number. Moreover, from the analysis, it is noted that the convectively heated surface significantly favors the local heat transport rate while low heat transfer rate is observed against an unsteady flow parameter. The temperature behaviour of the regular liquid over a stretchable sheet in the existence of constant heat flux condition is examined in Dutta and Roy^9^. In ordered to formulate the flow situation, they made certain assumption on the flow geometry and then portrayed the results for the flow regimes. The work was fascinating however it lacks the novelty of heat transfer in the nanofluids under more physical situation like magnetic field and thermal radiations.

The boundary layer flow analysis of a viscous fluid over a nonlinear stretchable surface is reported by Cortell^10^ in 2007. They described the model for two cases i.e., prescribed and constant surface temperature and plot the results against each case, respectively. They restricted their work only to conventional liquid however, this analysis could be prolonged to the various nanofluids under multiple flow conditions. The dynamics of the fluid by taking the influence of heat sink/source over a thermally heated surface is described in Bhattacharyya^11^. They also incorporated the phenomenon of fluid suction and injection from the surface and plotted the results by employing finite difference technique using quasi-linearization algorithm. The decrement in the thermal boundary layer is noticed against higher Prandtl number and stronger radiation effects. Further, rise in the heat transfer due to higher values of magnetic field and thermal radiations is observed.

The viscous dissipation is imperative in the analysis of fluid study and significantly alters its characteristics. These effects are analyzed in Partha et al.^12^ in the presence of buoyancy effects. Further, they discussed skin friction and heat transfer rate through computer generated graphical aids. The significant influences of convectively heated sheet and buoyancy effects on the fluid flow by considering Lorentz forces effects are explored and comprehensively discussed in^13,14^, respectively.

The study of hybrid-nanofluid by considering multilayered porous medium in the presence of internal sink is reported by Arasteh et al.^15^. They tackle the momentum and energy constitutive relations via two mathematical techniques and discussed the results comprehensively. The applications of heat transfer due to new generation of nanoliquids are widely strengthen their roots in many industrial and engineering fields such as mechanical engineering, to diagnose cancer cells, computer chips, manufacturing of home appliances, refrigeration, aerodynamics, manufacturing of solar aircraft parts, paint industries, heat exchangers and medical sciences. The role of imposed magnetic field, thermal radiations, internal heat source and nanoliquids effective empirical correlations cannot be ignored. Several recent significant studies in this regard are presented by different researchers^16–21^.

It is a reality that conventional liquids have low thermal conductivity and such fluids are not good heat conductor. The investigation of thermal transport mechanism over an unsteady surface is very significant. Such flows occur over bonnet of car, wings of air bus, solar thermal aircraft and over the surface of bullet. The study of heat transfer over unsteady surface is reported only for conventional liquid however; the work is extended for nanofluids. The used nanofluids (ND-H_2_O and Ag-H_2_O) has superior advantages over conventional liquids regarding heat transfer. The study of heat transfer over a permeable unsteady stretching surface for newtonian fluid is reported in Ene^22^. The authors modeled the problem by considering the impacts of viscous dissipation and prandtl effects and reported the results for the velocity and energy transport. From the study, it is observed that thermal performance of the fluid reduces due to higher prandtl effects. The analysis is interesting but it lacks the novelty enhanced heat transfer mechanism which has paramount importance for industrial and engineering purposes. Another study in this regard is reported in Elbashbeshy and Bazid^23^ for nonpermeable surface which is actually a special case of the study presented in Ene^22^ for conventional liquid. Therefore, the work published in^22,23^ could be prolonged for the study of nanofluids which is the demand of modern world. The main concerns of the study are to modify the existing models for nanofluids composed by ND and Ag nanoparticles and the comparison of thermal performance between them.

From eye bird investigation of the literature, it is realized that numerous studies are conducted to analyze the fluid flow for only conventional liquids that lack the novel heat transport in H_2_O comprising nano-diamond (ND) and Silver (Ag) nanomaterials. Therefore, the study is conducted to cover this innovative research gap in the field of nanofluids heat transfer enhancement. Thus, the nanofluid heat transfer model is formulated over an infinite semi-region and tackled numerically. The results are obtained against the physical parameters and significantly discussed through computer generated plots. To authenticate the colloidal analyses, a pictorial comparative analysis will be provided by using Mathematica 10.0 version.

## Mathematical modelling

### Problem statement and geometry

**Figure 1:**
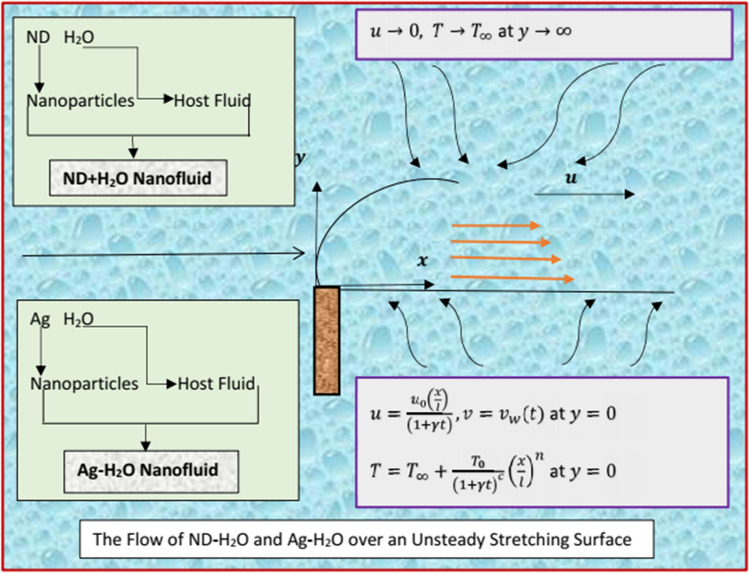
The Flow of nanodiamond-H_2_O and Silver-H_2_O.

Considered the flow of water suspended by nanodimaond and silver nanoparticles over an unsteady stretching sheet. The flow situation is configurated as two dimensional and unidirectional in Cartesian coordinates system. Let the nanofluid velocities along horizontal and vertical directions are prescribed by $u$ and $v$, respectively. Figure 1 depicting the flow of nanodiamond-H_2_O and Silver-H_2_O nanofluids over an unsteady stretching sheet:

### Governing model and similarity equations

In the view of above limitations, the law of conservation of mass, momentum and energy take the following form^22^:

1 $$ \frac{\partial u}{{\partial x}} + \frac{\partial v}{{\partial y}} = 0 $$

2 $$ \frac{\partial u}{{\partial t}} + u\frac{\partial u}{{\partial x}} + v\frac{\partial u}{{\partial y}} = \frac{{\mu_{nf} }}{{\rho_{nf} }}\left( {\frac{{\partial^{2} u}}{{\partial y^{2} }}} \right) $$

3 $$ \frac{\partial T}{{\partial t}} + u\frac{\partial T}{{\partial x}} + v\frac{\partial T}{{\partial y}} = \frac{{k_{nf} }}{{\left( {\rho C_{p} } \right)_{nf} }}\left( {\frac{{\partial^{2} T}}{{\partial y^{2} }}} \right) $$

In Eqs. (2) and (3), the effective dynamic viscosity, density, thermal conductivities and heat capacity are described by $\mu_{nf} , \rho_{nf} , k_{nf}$ and $\left( {\rho C_{p} } \right)_{nf}$, respectively. Moreover, the following effective models and thermophysical values are used:

$$ \rho_{nf} = \left( {\left( {1 - \phi } \right) + \phi \rho_{s} \left( {\rho_{f} } \right)^{ - 1} } \right)\rho_{f} $$

$$ \left( {\rho C_{p} } \right)_{nf} = \left( {\left( {1 - \phi } \right) + \phi \left( {\rho C_{p} } \right)_{s} \left( {\rho C_{p} } \right)_{f}^{ - 1} } \right)\left( {\rho C_{p} } \right)_{f} $$

$$ \mu_{nf} = \mu_{f} \left( {1 - \phi } \right)^{ - 2.5} $$

$$ k_{nf} = k_{s} \left( {\left( {k_{s} + 2k_{f} } \right) - 2\phi \left( {k_{f} - k_{s} } \right)} \right)\left( {\left( {k_{s} + 2k_{f} } \right) + \phi \left( {k_{f} - k_{s} } \right)} \right)^{ - 1} $$

The thermophysical values of ND, Ag and H_2_O are described in Table 1:

**Table 1:** Thermophysical values.

Characteristics	Density $\left( {\frac{\rho g}{{m^{3} }}} \right)$	Heat capacity $\left( {\frac{J}{Kg K}} \right)$	Thermal conductivity $\left( {W/mk} \right)$	Electrical conductivity $\left( {S/m} \right)$
Silver	10,500	234	425	6.21 × 10^6^
ND	3100	516	1000	34.84 × 10^6^
H_2_O	997.1	4179	0.613	0.005

The supported flow conditions at the surface and far from it are prescribed in the following way:

4 $$ u = u_{0} \left( {xl^{ - 1} } \right)\left( {1 + \gamma t} \right)^{ - 1} , v = v_{w} \left( t \right), T = T_{\infty } + T_{0} \left( {1 + \gamma t} \right)^{ - c} \left( {x^{n} l^{ - n} } \right)\,{\text{at}}\,{\text{the}}\,{\text{surface}} $$

5 $$ u \to 0 \,\, {\text{and}} \,\,T \to T_{\infty } ,\,{\text{far}}\,{\text{from}}\,{\text{the}}\,{\text{surface}} $$

In Eqs. (4) and (5), reference length is represented by $l$, $n$ and $c$. Further, $\gamma , T_{\infty , } u_{0}$ and $T_{0}$ are positive constants. Moreover, if $Re = lu_{0} \left( {\nu_{f} } \right)^{ - 1}$ and $Pr = \frac{{\nu_{f} }}{{k_{f} }}$, then the stream function $\varphi \left( {x,y} \right)$ described by the following expression:

6 $$ \varphi \left( {x,y} \right) = xl^{ - 1} \left( {\sqrt {Re}\, \left( {1 + \gamma t} \right)^{0.5} } \right)^{ - 1} F\left( \eta \right) $$

The velocity components from the stream function can be obtained by using the expressions $u = \frac{{\partial \varphi \left( {x,y} \right)}}{\partial y}$ and $v = - \frac{{\partial \varphi \left( {x,y} \right)}}{\partial x}$. The similarity transformation for the particular model is described as:

7 $$ \eta = \sqrt {Re}\,y\,\left( {l\left( {1 + \gamma t} \right)^{0.5} } \right)^{ - 1} $$

8 $$ T = T_{\infty } + T_{0} \,\left( {x^{n} l^{ - n} } \right)\theta \left( \eta \right)\left( {1 + \gamma t} \right)^{ - c} $$

In Eq. (8), the reference temperature is denoted by $T_{0}$.

The following velocity components along horizontal and vertical directions are attained from the given stream function:

9 $$ u = u_{0} xF^{\prime}\left( \eta \right)\left( {l\left( {1 + \gamma t} \right)} \right)^{ - 1} $$

10 $$ v_{w} = - u_{0} F\left( \eta \right)\left( {\sqrt {Re} \left( {1 + \gamma t} \right)^{0.5} } \right)^{ - 1} $$

Due to an unsteady flow, both $u$ and $v$ depend on time $t$. Now we compute the desired partial derivatives with respect to space and time variables from Eqs. (9) and (10).

11 $$ \frac{\partial u}{{\partial x}} = \frac{{u_{0} }}{{l\left( {1 + \gamma t} \right)}}F^{^{\prime}} \left( \eta \right) $$

12 $$ \frac{\partial u}{{\partial y}} = \frac{{u_{0} x\sqrt {\text{Re}} }}{{l^{2} \left( {1 + \gamma t} \right)^{3/2} }}F^{\prime \prime } \left( \eta \right) $$

13 $$ \frac{{\partial^{2} u}}{{\partial y^{2} }} = \frac{{u_{0} xRe}}{{l^{3} \left( {1 + \gamma t} \right)^{3/2} }}F^{\prime \prime \prime } \left( \eta \right) $$

14 $$ \frac{\partial u}{{\partial t}} = - u_{0} x\gamma \left( {l\left( {1 + \gamma t} \right)^{2} } \right)^{ - 1} F^{\prime } \left( \eta \right) - \frac{{u_{0} x\gamma y\sqrt {Re} }}{{2l^{2} \left( {1 + \gamma t} \right)^{\frac{5}{2}} }}F^{\prime \prime } \left( \eta \right) $$

15 $$ \frac{\partial T}{{\partial t}} = - T_{0} c\left( \frac{x}{l} \right)^{c} \gamma \left( {1 + \gamma t} \right)^{{ - \left( {c + 1} \right)}} \theta \left( \eta \right) - \frac{{T_{0} \left( \frac{x}{l} \right)^{c} \gamma y\sqrt {Re} }}{{2l\left( {1 + \gamma t} \right)^{{c + \frac{3}{2}}} }}\theta^{^{\prime}} \left( \eta \right) $$

16 $$ \frac{\partial T}{{\partial x}} = \frac{{nT_{0} \left( \frac{x}{l} \right)^{n - 1} }}{{l\left( {1 + \gamma t} \right)^{c} }}\theta \left( \eta \right) $$

17 $$ \frac{\partial T}{{\partial y}} = \frac{{T_{0} \left( \frac{x}{l} \right)^{c} \sqrt {Re} }}{{l\left( {1 + \gamma t} \right)^{{c + \frac{1}{2}}} }}\theta^{^{\prime}} \left( \eta \right) $$

18 $$ \frac{{\partial^{2} T}}{{\partial y^{2} }} = \frac{{T_{0} \left( \frac{x}{l} \right)^{c} \sqrt {Re} }}{{l^{2} \left( {1 + \gamma t} \right)^{c + 1} }}\theta^{\prime \prime } \left( \eta \right) $$

By using Eqs. (11–14) and defined effective nanofluid models in Eq. (2) and after performing necessary calculation, the following dimensionless momentum equation is attained:

19 $$ F^{\prime\prime\prime} + \frac{{\left( {1 - \phi } \right)^{2.5} }}{{\left( {1 - \phi + \phi \rho_{s} \rho_{f}^{ - 1} } \right)^{ - 1} }}\left( {FF^{\prime \prime } - F^{^{\prime}2} + A_{1} \left( {F^{\prime } + 0.5\eta F^{\prime \prime } } \right)} \right) = 0 $$

where $A_{1}$ represents the unsteadiness number and defined by the formula $A_{1} = \gamma lu_{0}^{ - 1}$. Now using the Eqs. (15–18), effective thermal conductivity and the appropriate velocity components in Eq. (3), the following dimensionless version of the energy equation is obtained:

20 $$ \theta^{\prime \prime } + \frac{{\left( {1 - \phi } \right) + \phi \left( {\rho C_{p} } \right)_{s} \left( {\rho C_{p} } \right)_{f}^{ - 1} }}{{\left( {\left( {k_{s} + 2k_{f} } \right) - 2\phi \left( {k_{f} - k_{s} } \right)} \right)\left( {\left( {k_{s} + 2k_{f} } \right) + \phi \left( {k_{f} - k_{s} } \right)} \right)^{ - 1} }}\left( {PrA_{1} \left( {c\theta + 0.5\eta \theta^{\prime } } \right) + \Pr \left( {n\theta F^{\prime } + \theta^{\prime } F} \right)} \right) = 0 $$

In Eq. (20), $Pr$ is the Prandtl number and described as $Pr = \nu_{f} /k_{f}$.

The boundary conditions described in Eqs. (4) and (5) reduced in the following version after using the appropriate velocity components and necessary calculation:

21 $$ F\left( {\eta_{ = 0} } \right) = f_{w} , F^{\prime } \left( {\eta_{ = 0} } \right) = 1 \,\,and \,\, F^{\prime } \left( {\eta_{ = \infty } } \right) = 0 $$

22 $$ \theta \left( {\eta_{ = 0} } \right) = 1 \,\,and \,\, \theta \left( {\eta_{ = \infty } } \right) = 0 $$

### Mathematical investigation of the model

The nanofluid flow model described in Eqs. (19) and (20) is highly nonlinear over semi-infinite region. Therefore, it is inappropriate to handle the model by using exact mathematical techniques. Thus, keeping in view the nature of the nanofluid model, a numerical technique known as RK coupled with shooting algorithm (built-in in Mathematica 10.0) is used to handle the model. In this case, firstly the model is transformed into a system of first order ODEs and then a built-in algorithm is implemented for further mathematical computation. The particular model reduced into a system of ODEs in the following way. Let us introduce the following transformations:

23 $$ \chi_{1} = F, \chi_{2} = F^{\prime } \,and\, \chi_{3} = F^{\prime \prime } $$

24 $$ \chi_{4} = \theta and \chi_{5} = \theta^{\prime } $$

From Eqs. (23) and (24), we obtained

25 $$ \chi_{1}^{\prime } = F^{\prime } , \,\chi_{2}^{\prime } = F^{\prime \prime } \, and\, \chi_{3}^{\prime } = F^{\prime \prime \prime } $$

26 $$ \chi_{4}^{\prime } = \theta^{^{\prime} } \, and\, \chi_{5}^{\prime } = \theta^{\prime \prime } $$

The model Eqs. (19) and (20) can be rearranged in the following fashion:

27 $$ F^{\prime \prime \prime } = - \left( {\frac{{\left( {1 - \phi } \right)^{2.5} }}{{\left( {1 - \phi + \phi \rho_{s} \rho_{f}^{ - 1} } \right)^{ - 1} }}\left( {FF^{\prime \prime } - F^{^{\prime}2} + A_{1} \left( {F^{\prime } + 0.5\eta F^{\prime \prime } } \right)} \right)} \right) $$

28 $$ \theta^{\prime \prime } = - \left( {\frac{{\left( {1 - \phi } \right) + \phi \left( {\rho C_{p} } \right)_{s} \left( {\rho C_{p} } \right)_{f}^{ - 1} }}{{\left( {\left( {k_{s} + 2k_{f} } \right) - 2\phi \left( {k_{f} - k_{s} } \right)} \right)\left( {\left( {k_{s} + 2k_{f} } \right) + \phi \left( {k_{f} - k_{s} } \right)} \right)^{ - 1} }}\left( {\Pr A_{1} \left( {c\theta + 0.5\eta \theta^{\prime } } \right) + \Pr \left( {n\theta F^{\prime } + \theta^{\prime } F} \right)} \right)} \right) $$

In the view of Eqs. (27) and (28), $\chi_{3}^{\prime }$ and $\chi_{5}^{\prime }$ reduced into the following form:

29 $$ \chi_{3}^{\prime } = - \left( {\frac{{\left( {1 - \phi } \right)^{2.5} }}{{\left( {1 - \phi + \phi \rho_{s} \rho_{f}^{ - 1} } \right)^{ - 1} }}\left( {FF^{\prime \prime } F^{\prime 2} + A_{1} \left( {F^{\prime } + 0.5\eta F^{\prime \prime } } \right)} \right)} \right) $$

30 $$ \chi_{5}^{\prime } = - \left( {\frac{{\left( {1 - \phi } \right) + \phi \left( {\rho C_{p} } \right)_{s} \left( {\rho C_{p} } \right)_{f}^{ - 1} }}{{\left( {\left( {k_{s} + 2k_{f} } \right) - 2\phi \left( {k_{f} - k_{s} } \right)} \right)\left( {\left( {k_{s} + 2k_{f} } \right) + \phi \left( {k_{f} - k_{s} } \right)} \right)^{ - 1} }}\left( {\Pr A_{1} \left( {c\theta + 0.5\eta \theta^{\prime } } \right) + \Pr \left( {n\theta F^{\prime } + \theta^{\prime } F} \right)} \right)} \right) $$

Which further rearranged in the following manner:

31 $$ \chi_{3}^{\prime } = - \left( {\frac{{\left( {1 - \phi } \right)^{2.5} }}{{\left( {1 - \phi + \phi \rho_{s} \rho_{f}^{ - 1} } \right)^{ - 1} }}\left( {\chi_{1} \chi_{3} - \chi_{2}^{2} + A_{1} \left( {\chi_{2} + 0.5\eta \chi_{3} } \right)} \right)} \right) $$

32 $$ \chi_{5}^{\prime } = - \left( {\frac{{\left( {1 - \phi } \right) + \phi \left( {\rho C_{p} } \right)_{s} \left( {\rho C_{p} } \right)_{f}^{ - 1} }}{{\left( {\left( {k_{s} + 2k_{f} } \right) - 2\phi \left( {k_{f} - k_{s} } \right)} \right)\left( {\left( {k_{s} + 2k_{f} } \right) + \phi \left( {k_{f} - k_{s} } \right)} \right)^{ - 1} }}\left( {PrA_{1} \left( {c\chi_{4} + 0.5\eta \chi_{5} } \right) + \Pr \left( {n\chi_{4} \chi_{2} + \chi_{5} \chi_{1} } \right)} \right)} \right) $$

After this, Mathematica 10.0 is used to tackle the particular model and for the graphical results.

### Graphical results with discussion

The flow quantities significantly alter the flow characteristics over the desired domain. Therefore, this section is devoted to examine the behaviour of dimensionless velocity profile ($F^{\prime } \left( \eta \right)$) and temperature profile ($\theta \left( \eta \right)$) of ND-H_2_O and Ag-H_2_O nanofluids by varying the flow parameters. Further, the velocity and the thermal behaviour of The nanofluids examine in first and second subsection, respectively. Further, to authenticate the analysis, a pictorial comparative analysis will be made in third subsection under certain assumptions on the flow models.

### The velocity $F^{\prime}\left( \eta \right)$ distribution

#### The Nanofluid velocity $F^{\prime}\left( \eta \right)$ against $f_{w}$

**Figure 2:**
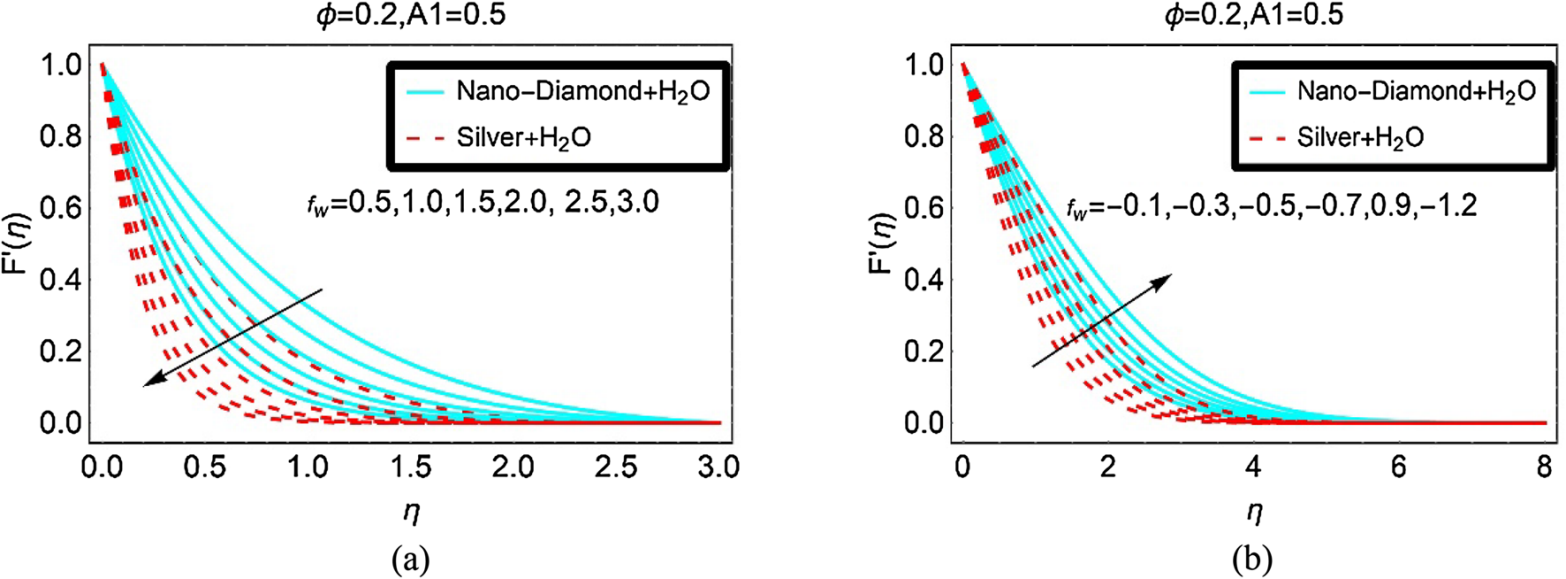
The velocity against (**a**) Suction and (**b**) Injection.

Figure 2 illustrated the behaviour of ND-H_2_O and Ag-H_2_O nanofluids velocity $F^{\prime } \left( \eta \right)$ against multiple values of suction and injection, respectively. Figure 2a demonstrates that the velocity $F^{\prime } \left( \eta \right)$ for both the nanofluids declines due to the stronger suction effects at the sheet. Physically, more nanofluid particles stuck at the surface due to suction effects as a result the fluid velocity $F^{\prime } \left( \eta \right)$ decreases. For Ag-H_2_O, the motion declines abruptly because the nanofluid becomes denser due to its high-density comparative to ND nanoparticles. The velocity $F^{\prime } \left( \eta \right)$ of ND-H_2_O drops slowly and low density of ND is responsible for this behaviour of the fluid velocity $F^{\prime } \left( \eta \right)$.

The influences of injecting fluid from the surface over the velocity $F^{\prime } \left( \eta \right)$ of ND-H_2_O and Ag-H_2_O are captured in Fig. 2b. The favorable effects of injection on the velocity $F^{\prime } \left( \eta \right)$ of the nanofluids are examined. Physically, the fluid particles left the sheet surface due to injection therefore, the momentum of the fluid particles rises that ultimately leads to upturns in the fluid motion. further, the intermolecular forces become weaker in the composition of ND-H_2_O due to its lower density and the fluid particles move freely over the desired domain. Due to which the velocity of ND-H_2_O rises abruptly comparative to Ag-H_2_O nanofluid.

#### The nanofluid velocity $F^{\prime } \left( \eta \right)$ against $A_{1}$

**Figure 3:**
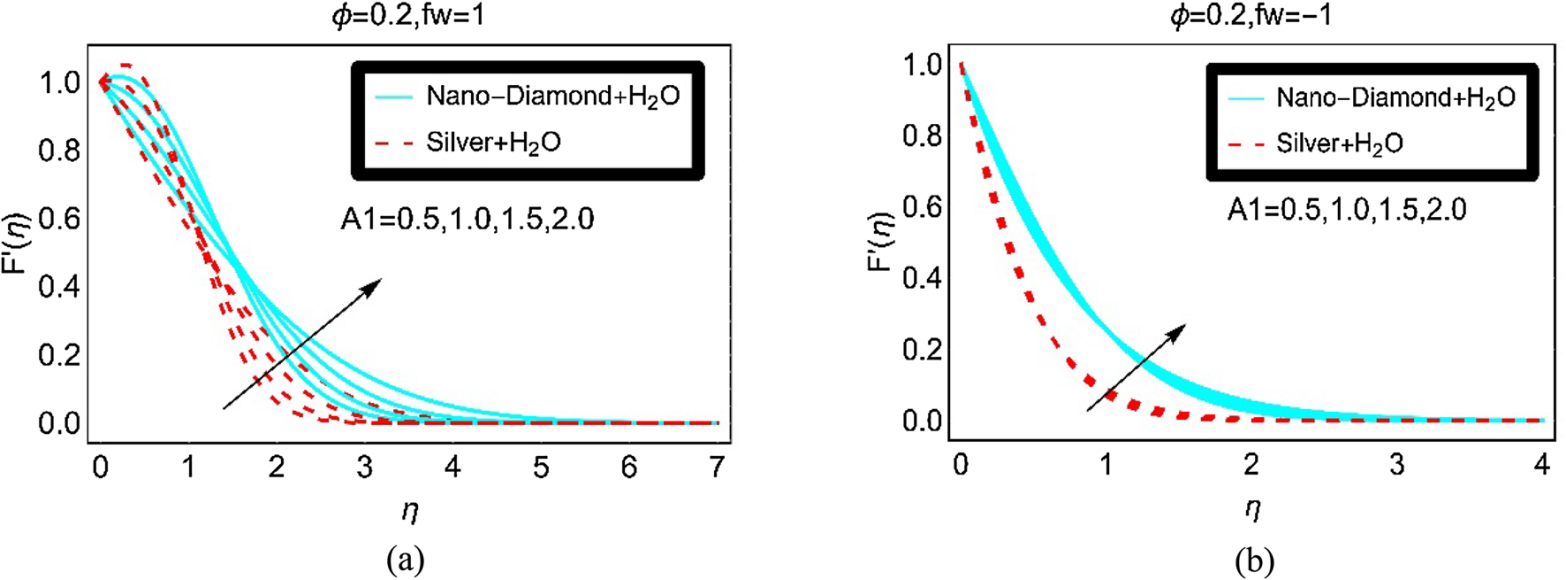
The velocity against (**a**) Suction and (**b**) Injection for varying A_1_.

The effects of unsteady parameter $A_{1}$ on the nanofluid velocities at fixed suction and injection are elaborated in Fig. 3a and b, respectively. The results are captured for both ND-H_2_O and Ag-H_2_O nanofluids. From the plotted results, it is explored that the velocity $F^{\prime } \left( \eta \right)$ of the nanofluids changes dually over the region of interest. For higher unsteady parameter, the velocity $F^{\prime } \left( \eta \right)$ rapidly increases. Near the vicinity of the surface, the fluids motion drops and then after $\eta > 1$, it starts increases. The increment in the fluids velocity $F^{\prime } \left( \eta \right)$ gradually slowdown and finally it vanishes asymptotically beyond $\eta > 5$. On the other hand, almost negligible variations in the fluid velocity $F^{\prime } \left( \eta \right)$ for multiple values of A_1_ are observed by keeping $f_{w} = - 1$. These effects are elucidated in Fig. 3b.

**Figure 4:**
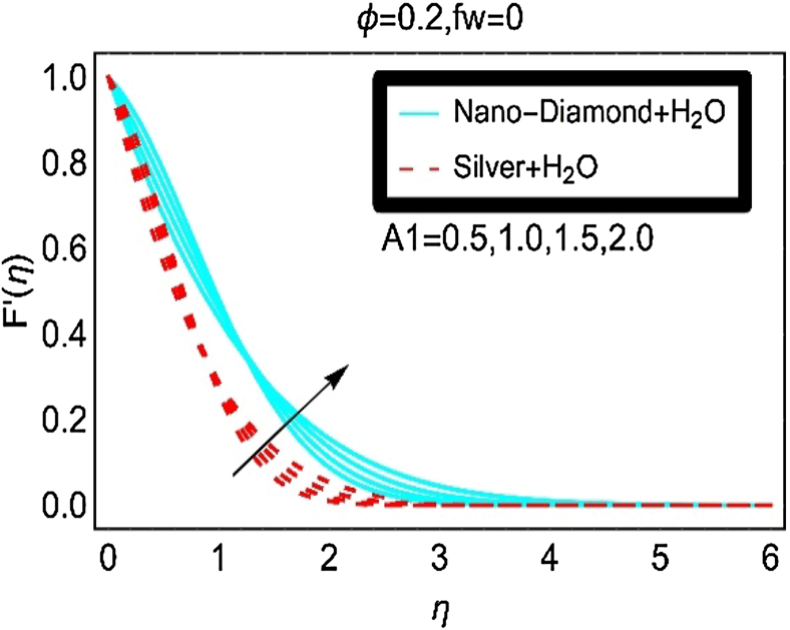
The velocity against $fw = 0$ for varying A_1_.

The nanofluids velocity $F^{\prime } \left( \eta \right)$ due to varying unsteady parameter and in the absence of suction/injection is plotted in Fig. 4. The velocity $F^{\prime } \left( \eta \right)$ of the nanofluids is quite rapid in this case comparative to the fluid velocity $F^{\prime } \left( \eta \right)$ in Fig. 3b. Moreover, slow increment in the velocity $F^{\prime } \left( \eta \right)$ for Silver-H_2_O nanofluid is observed due to high density the nanoparticles.

### Temperature distribution $\theta \left( \eta \right)$

#### The nanofluids temperature $\theta \left( \eta \right)$ against $f_{w}$

The effective thermal physical values of the nanomaterials are key factors in the analysis of the nanofluids regarding the heat transport mechanism. These values significantly alter the fluids thermal characteristics. Therefore, temperature profile $\theta \left( \eta \right)$ of ND-H_2_O and Ag-H_2_O under varying multiple parameters are decorated in this subsection.

**Figure 5:**
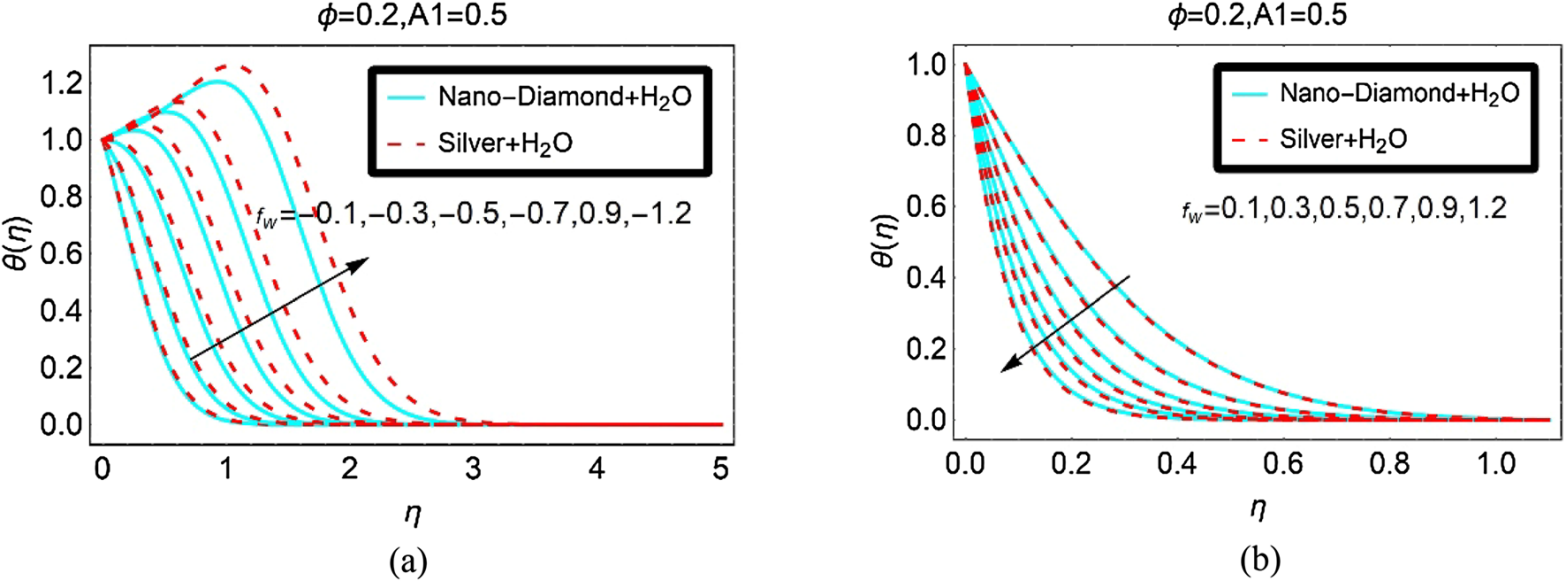
The Temperature against (**a**) Injection and (**b**) Suction.

Figure 5 demonstrated the temperature $\theta \left( \eta \right)$ for injection and suction, respectively. It is observed that the temperature $\theta \left( \eta \right)$ of the nanofluids rises significantly due to more injecting fluid. Physically, the fluid motion rises rapidly due to suction which lead to increment in the kinetic energy of the fluid particles. Due to high kinetic energy, the particles collision rises. As a result, the temperature $\theta \left( \eta \right)$ increases abruptly. Near the surface, the temperature $\theta \left( \eta \right)$ boosts rapidly due to stronger injection effects and it asymptotically vanishes beyond $\eta \ge 3$. These effects are painted in Fig. 5a for both nanofluids. The behaviour of $\theta \left( \eta \right)$ against suction fluid is portrayed in Fig. 5b. From this, it can be examined that the temperature $\theta \left( \eta \right)$ declines due to higher values of the suction parameter.

#### The nanofluids temperature $\theta \left( \eta \right)$ against $A_{1}$

**Figure 6:**
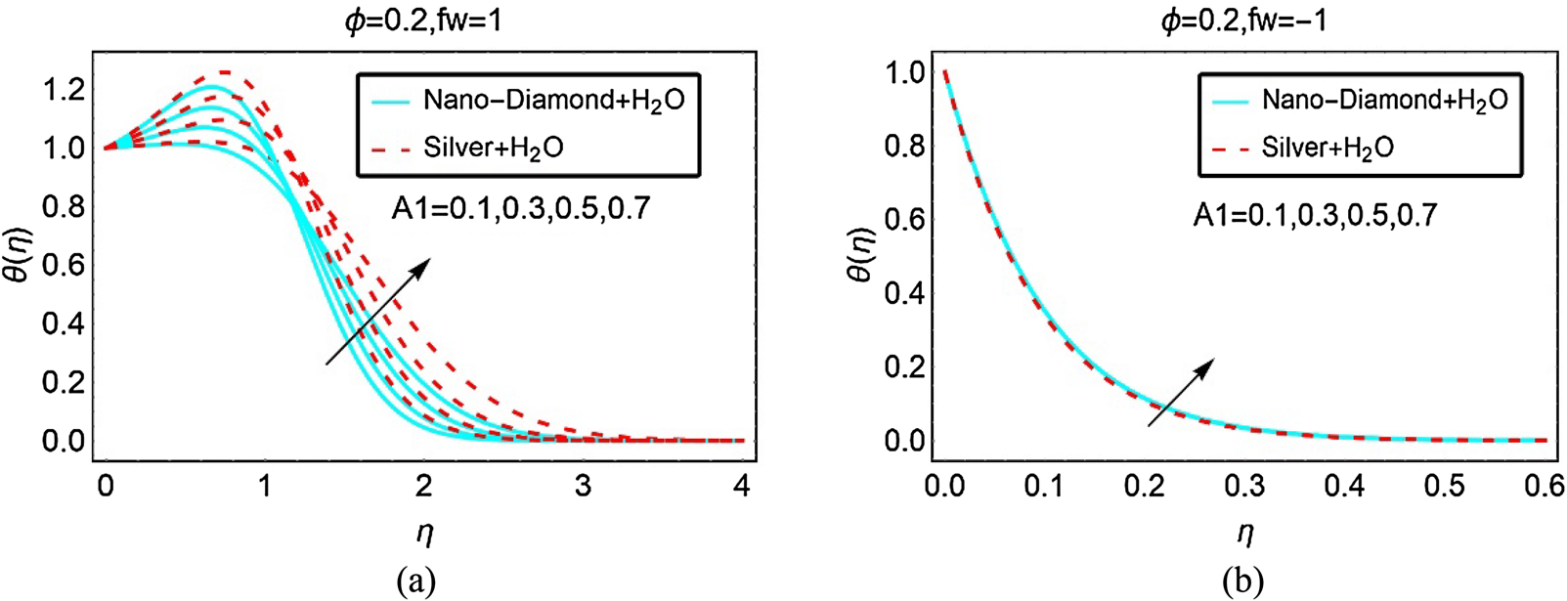
The Temperature against (**a**) Suction and (**b**) Injection for varying A_1_.

**Figure 7:**
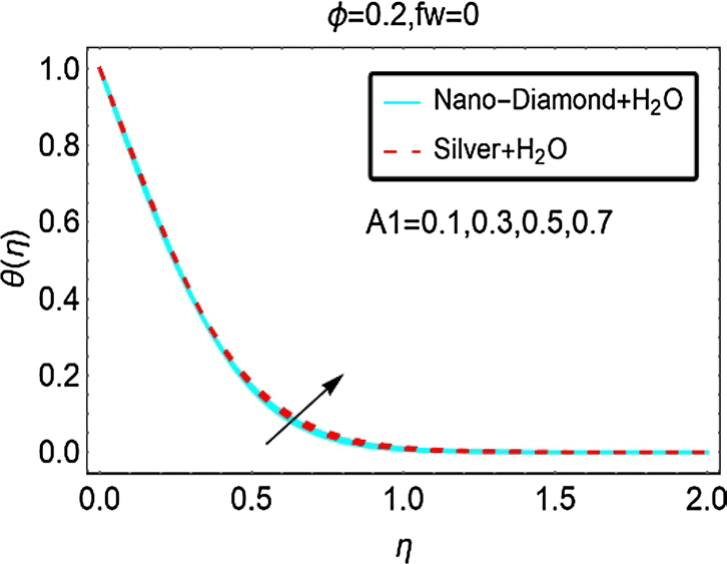
The Temperature against $f_{w} = 0$ for varying A_1_.

The role of unsteady parameter $A_{1}$ on the temperature $\theta \left( \eta \right)$ of ND-H_2_O and Ag-H_2_O is illustrated in Figs. 6 and 7 for suction, injection and in the absence of suction/injection, respectively. It is noted that the unsteady parameter significantly intensifies the nanofluids temperature $\theta \left( \eta \right)$ at constant suction. The temperature $\theta \left( \eta \right)$ significantly rises for Silver-H_2_O nanofluid in the region $1 < \eta < 3$. The temperature $\theta \left( \eta \right)$ of ND-H_2_O is also increasing however, the increment is quite slow comparative to Ag-H_2_O. These influences are pictured in Fig. 6a. Further, rest of the Figs. 6b and 7 indicates almost inconsequential variations in the temperature $\theta \left( \eta \right)$ due to higher unsteady effects.

### Thermophysical characteristics against $\phi$

**Figure 8:**
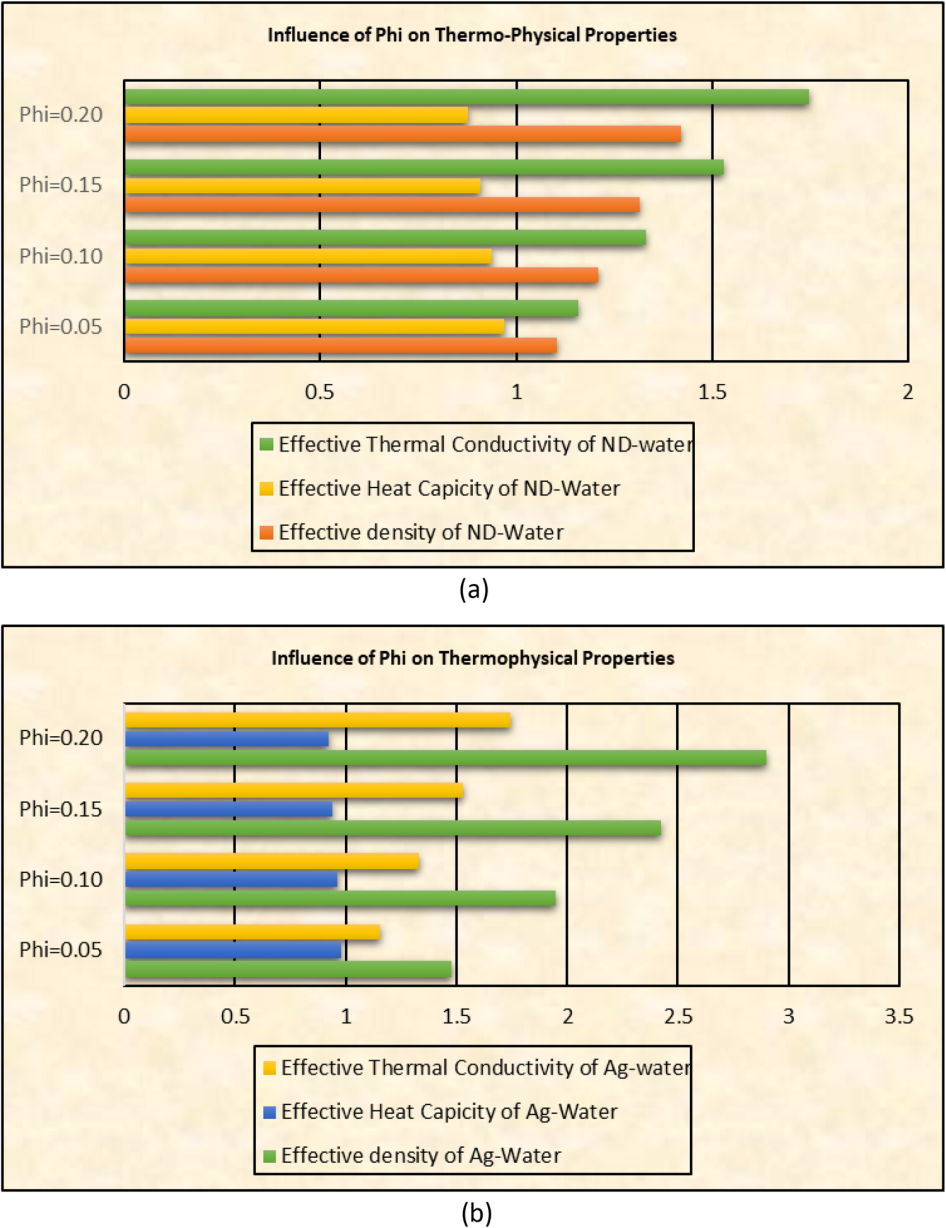
Influence of $\phi$ on thermophysical values of (**a**) ND-H_2_O and (**b**) Ag-H_2_O.

The volume fraction ($\phi$) of the nanomaterials effectively alters thermophysical values which play vibrant role in the heat transport mechanism in the nanofluids. These values for effective density, dynamic viscosity and thermal conductivity against $\phi$ are elaborated in Figs. 8a and b for under consideration nanofluids, respectively. From the keen view, it is noticed that the thermal conductivity of Ag-H_2_O is greater than the ND-H_2_O. therefore, Ag-H_2_O nanofluid is better conductor and has high heat transport characteristics. Similarly, effective density and heat capacity enhances by augmenting the volumetric fraction of the nanomaterials.

### Authentication of the study

The presented work is the modified version of the studies reported in^22,23^. Therefore, to validate the present analysis, graphical and tabular comparison is made under certain assumptions ($\phi = 0$ for graphical comparison and $f_{w} = 0,\phi = 0$ for tabular comparison) on the current nanofluid model.

**Figure 9:**
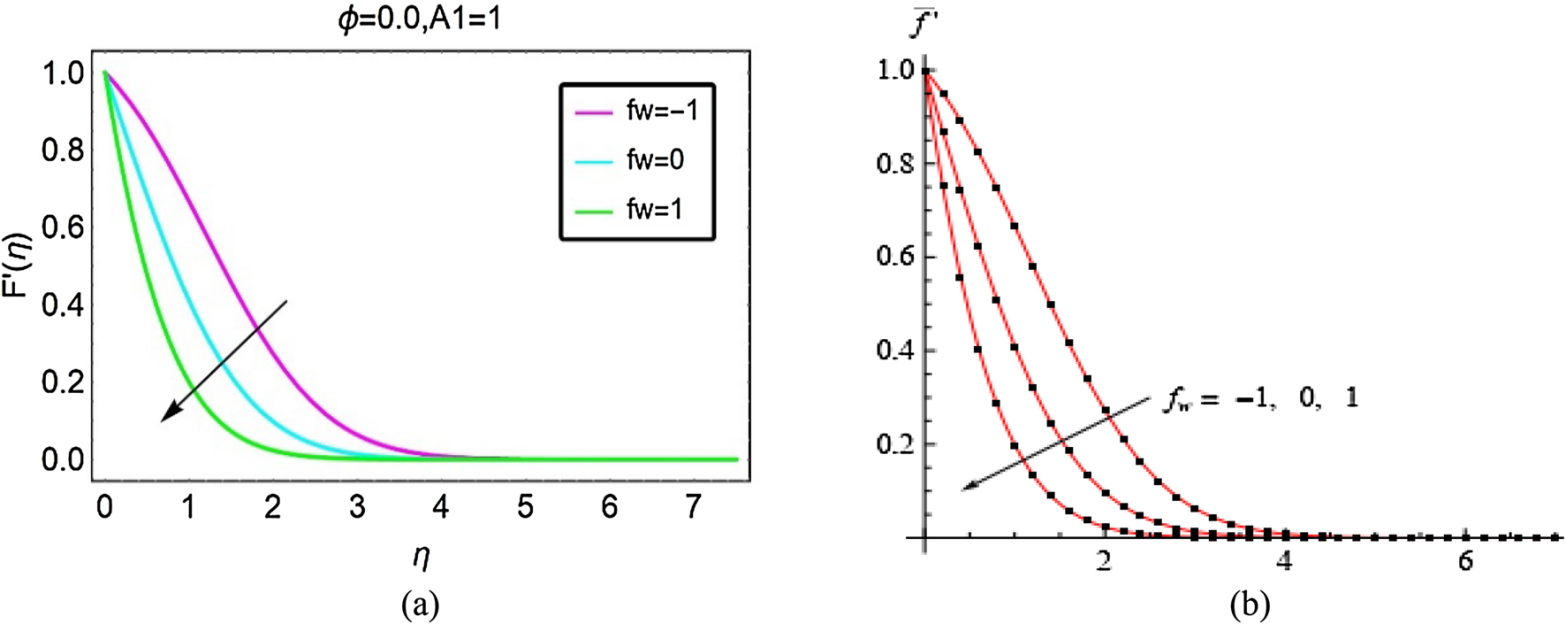
The pictorial comparative analysis for $F^{\prime } \left( \eta \right)$^22^.

From Fig. 9, it is cleared that the present velocity profile by taking $\phi = 0$ in the model is similar to the velocity field (conventional fluid) examined in^22^ which evidences the current analysis is valid. Also, from Table 2, the present results are more accurate with previous study^23^. Therefore, more credibility of the presented work is subject to the below provided comparison.

**Table 2:** Comparison of present results with previously published work.

$A_{1}$	0.8	1.2	2.0
Present	1.32342	1.42353	1.61214
Elbashbeshy and Bazid^23^	1.3321	1.4691	1.7087

### Major findings

The investigation of enhanced thermal transport over an unsteady sheet for nanofluids is not yet reported which is a major research gap. A newly developed generation of conventional liquids known as Nanofluids attained much popularity of the researchers and engineers due to their high thermal performance rate. Therefore, the analysis of thermal transport mechanism for ND-H_2_O and Ag-H_2_O is organized over an unsteady sheet. Such type of flows occurs over car bonnet, solar thermal aircraft and over the surface of bullet. Therefore, a flow problem is modeled for ND-H_2_O and Ag-H_2_O nanofluids and examined the influences of ingrained parameters on the heat transfer mechanism over an unsteady surface. From the analysis, it is concluded that the used nanofluids are better heat conductor and would be beneficial for industrial and engineering purposes than conventional liquids. Finally, to check the credibility of the study graphical and tabular comparison is provided and found excellent agreement with previous work.

## Data Availability

The authors declared no additional data for this manuscript.

## References

[1] Elgazery NS (2019). Nanofluids flow over a permeable unsteady stretching surface with non-uniform heat source/sink in the presence of inclined magnetic field. J. Egypt. Math. Soc..

[2] Nasir S, Shah Z, Islam S, Bonyah E, Gul T (2019). Darcy Forchheimer nanofluid thin film flow of SWCNTs and heat transfer analysis over an unsteady stretching sheet. AIP Adv..

[3] Kebede T, Haile E, Awgichew G, Walelign T (2020). Heat and mass transfer in unsteady boundary layer flow of williamson nanofluids. J. Appl. Math..

[4] Sreedevi P, Reddy PS, Chamkha A (2020). Heat and mass transfer analysis of unsteady hybrid nanofluid flow over a stretching sheet with thermal radiation. SN Appl. Sci..

[5] Das K, Duari PR, Kundu PK (2014). Nanofluid flow over an unsteady stretching surface in presence of thermal radiation. Alex. Eng. J..

[6] Prasannakumara BC, Gireesha BJ, Krishnamurthy MR, Gorla RSR (2017). Unsteady boundary layer flow and convective heat transfer of a fluid particle suspension with nanoparticles over a stretching surface. J. Model. Mech. Mater..

[7] Bhattacharyya K, Layek GC (2014). Magnetohydrodynamic boundary layer flow of nanofluid over an exponentially stretching permeable sheet. Phys. Res. Int..

[8] Mansur S, Ishak A (2016). Unsteady boundary layer flow of a nanofluid over a stretching/shrinking sheet with a convective boundary condition. J. Egyptian Math. Soc..

[9] Dutta BK, Roy P (1985). Temperature field in flow over a stretching sheet with uniform heat flux. Int. Commun. Heat Mass Transfer.

[10] Cortell R (2007). Viscous flow and heat transfer over a nonlinearly stretching sheet. Appl. Math. Comput..

[11] Bhattacharyya K (2011). Effects of radiation and heat source/sink on unsteady MHD boundary layer flow and heat transfer over a shrinking sheet with suction/injection. Front. Chem. Sci. Eng..

[12] Partha MK, Murthy PVSN, Rajasekhar GP (2005). Effect of viscous dissipation on the mixed convection heat transfer from an exponentially stretching surface. Heat Mass Transf..

[13] Makinde OD, Aziz A (2011). Boundary layer flow of a nanofluid past a stretching sheet with a convective boundary condition. Int. J. Therm. Sci..

[14] Makinde OD, Khan WA, Khan ZH (2013). Buoyancy effects on MHD stagnation point flow and heat transfer of a nanofluid past a convectively heated stretching/shrinking sheet. Int. J. Heat Mass Transf..

[15] Arasteh H, Mashayekhi R, Toghraie D, Karimipour A, Bahiraei M, Rahbari A (2019). Optimal arrangements of a heat sink partially filled with multilayered porous media employing hybrid nanofluid. J. Therm. Anal. Calorim..

[16] Gheynani R, Akbari OA, Zarringhalam M, Shabani GAS, Alnaqi AA, Goodarzi M, Toghraie D (2019). Investigating the effect of nanoparticles diameter on turbulent flow and heat transfer properties of non-Newtonian carboxymethyl cellulose/CuO fluid in a microtube. Int. J. Numer. Meth. Heat Fluid Flow.

[17] Oveissi S, Eftekhari SA, Toghraie D (2016). Longitudinal vibration and instabilities of carbon nanotubes conveying fluid considering size effects of nanoflow and nanostructure. Phys. E.

[18] Faridzadeh MR, Toghraie DS, Niroomand A (2014). Analysis of laminar mixed convection in an inclined square lid-driven cavity with a nanofluid by using an artificial neural network. Heat Transfer Res..

[19] Shahsavar A, Godini A, Sardari PT, Toghraie D, Salehipour H (2019). Impact of variable fluid properties on forced convection of Fe_3_O_4_/CNT/water hybrid nanofluid in a double-pipe mini-channel heat exchanger. J. Therm. Anal. Calorim..

[20] Toghraie D, Sina N, Jolfaei NA, Hajian M, Afrand M (2019). Designing an Artificial Neural Network (ANN) to predict the viscosity of Silver/Ethylene glycol nanofluid at different temperatures and volume fraction of nanoparticles. Phys. A Stat. Mech. Appl..

[21] Barnoon P, Toghraie D, Eslami F, Mehmandoust B (2019). Entropy generation analysis of different nanofluid flows in the space between two concentric horizontal pipes in the presence of magnetic field: Single-phase and two-phase approaches. Comput. Math. Appl..

[22] Ene RD, Marinca V, Marinca VB (2016). Viscous flow and heat transfer over an unsteady stretching surface. Open Phys..

[23] Elbashbeshy EMA, Bazid MAA (2004). Heat transfer over an unsteady stretching surface. Heat Mass Transfer.

